# Intra-Strain Genetic Variation of Platyfish (*Xiphophorus maculatus*) Strains Determines Tumorigenic Trajectory

**DOI:** 10.3389/fgene.2020.562594

**Published:** 2020-10-06

**Authors:** Yuan Lu, Taryn J. Olivas, Mikki Boswell, William Boswell, Wes C. Warren, Manfred Schartl, Ronald B. Walter

**Affiliations:** ^1^Xiphophorus Genetic Stock Center, Texas State University, San Marcos, TX, United States; ^2^Department of Cell Biology, Yale University School of Medicine, New Haven, CT, United States; ^3^Bond Life Sciences Center, University of Missouri, Columbia, MO, United States; ^4^Developmental Biochemistry, Theodor-Boveri-Institute, Biozentrum, University of Würzburg, Würzburg, Germany

**Keywords:** *Xiphophorus*, polymorphism, comparative genomics, inter-strain genetic variants, mutation cluster

## Abstract

*Xiphophorus* interspecies hybrids represent a valuable model system to study heritable tumorigenesis, and the only model system that exhibits both spontaneous and inducible tumors. Types of tumorigenesis depend on the specific pedigree of the parental species, *X. maculatus*, utilized to produce interspecies hybrids. Although the ancestors of the two currently used *X. maculatus* parental lines, Jp163 A and Jp163 B, were originally siblings produced by the same mother, backcross interspecies hybrid progeny between *X. hellerii* and *X. maculatus* Jp163 A develop spontaneous melanoma initiating at the dorsal fin due to segregation of an oncogene and a regulator encoded by the *X. maculatus* genome, while the backcross hybrid progeny with *X. hellerii* or *X. couchianus* and Jp163 B exhibit melanoma on the flanks of their bodies, especially after treatment with ultraviolet light. Therefore, dissecting the genetic differences between these two closely related lines may lead to better understanding of functional molecular differences associated with tumorigenic mechanisms. For this purpose, comparative genomic analyses were undertaken to establish genetic variants between these two *X. maculatus* lines. Surprisingly, given the heritage of these two fish lines, we found genetic variants are clustered together in select chromosomal regions. Among these variants are non-synonymous mutations located in 381 genes. The non-random distribution of genetic variants between these two may highlight ancestral chromosomal recombination patterns that became fixed during subsequent inbreeding. Employing comparative transcriptomics, we also determined differences in the skin transcriptional landscape between the two lines. The genetic differences observed are associated with pathways highlighting fundamental cellular functions including inter-cellular and microenvironment-cellular interactions, and DNA repair. These results collectively lead to the conclusion that diverged functional genetic baselines are present between Jp163 A and B strains. Further, disruption of these fixed genetic baselines in the hybrids may give rise to spontaneous or inducible mechanisms of tumorigenesis.

## Introduction

Melanoma is a devastating disease with continuously growing incidence despite a decreasing trend of cancer incidences for most cancer types over the past few decades. Risk factors include genetic background (i.e., hereditary/familial history, congenital nevi syndromes, skin types), age, sex, immune status, and UV exposure. Variability in genetic background in humans is known to determine susceptibility to melanoma, and possibly body sites (Dennis, 1999; Potrony et al., 2015). However, animal models that produce both heritable and induced melanoma, for dissection of genetic interactions underlying different tumorigenic mechanisms, or assessment of genetic vs. environmental contributions to disease, is very rare. Melanoma development in *Xiphophorus* is similar to that of humans at the histological, transcriptome, and signaling pathway levels (Potrony et al., 2015; Lu et al., 2018). These attributes render the *Xiphophorus* system as the only model wherein one may study genetic interactions underlying divergent melanoma-genic mechanisms.

**FIGURE 1:**
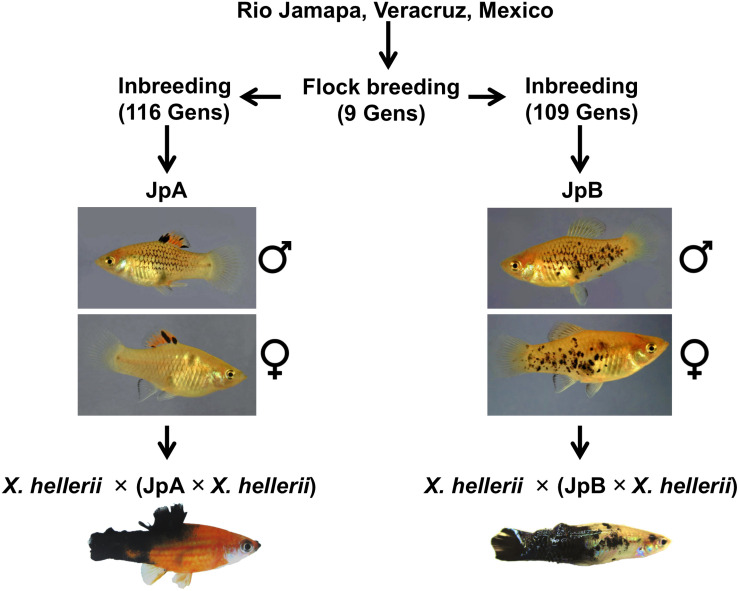
*Xiphophorus maculatus* A and Jp163 B strains. The ancestors of JpA and JpB strains were siblings from a single *X. maculatus* collected in the Jamapa river near Veracruz, Mexico. JpA and JpB were separated into two strains at the *Xiphophorus* Genetic Stock Center. Both strains have been inbred (i.e., brother-sister mating) for over 110 generations. JpA and JpB strains are different by their macromelanophore pigmentation patterns, with JpA exhibiting *Spot dorsal* (*Sd*) and JpB having the *Spot side* (*Sp*) pattern. Approximately 50% of backcross interspecies hybrid between JpA and *X. hellerii* that inherited the parental pigmentation pattern produce spontaneous tumor on dorsal fin, tail fin that invades to muscle. The backcross interspecies hybrid between JpA and *X. hellerii* produce spontaneous tumor on dorsal fin, tail fin that invades to muscle. Approximately 5% of backcross interspecies hybrid between JpB and *X. hellerii* that inherited the parental pigmentation pattern develop tumor on side of the body.

*Xiphophorus* fish, commonly known as platyfish and swordtails, comprise a genus consisting of 26 species of live-bearing fishes commonly found in Mexico, Central and South America. *Xiphophorus maculatus* represents a long-standing genetic model that has been adopted to study cancer etiology. The most commonly used *X. maculatus* lines, Jp163 A (JpA) and Jp163 B (JpB), are descendants of siblings derived from a single brood from an *X. maculatus* female collected in the Rio Jamapa, Veracruz, Mexico in 1939 (Walter et al., 2006). This brood produced fish that exhibited very different pigmentation patterns: The JpA line is characterized by a *spotted dorsal* (*Sd*) pigmentation pattern, while JpB is characterized by a *spotted side* (*Sp*) pigmentation pattern (Figure 1). After ≈9 generations of intercrossing (i.e., flock mating), the JpA and JpB lines were separated as distinct pedigrees and have since been maintained as inbred lines (i.e., brother sister matings). These two lines are currently in their 116th (JpA) and 109th (JpB) inbred generation and are available from the *Xiphophorus* Genetic Stock Center (Walter et al., 2019).

The species richness of *Xiphophorus* provides a unique model system that allows production of fertile interspecies hybrids between species that diverged from one another several million years ago (Cui et al., 2013; Jones et al., 2013). This allows identification of incompatible genes underlying negative epistatic interactions. One such interaction is produced by crossing *X. maculatus* JpA to *X. hellerii*, followed by backcrossing the F_1_ interspecies hybrid to *X. hellerii* (Anders, 1967). This model serves as one of the only two vertebrate examples of Bateson–Dobzhansky–Muller (BDM) genetic incompatibility, and also shows that tumorigenesis is a mechanism that may lead to decreased fitness in hybrids (Adam et al., 1993; Noor, 2003; Pennisi, 2006; Maheshwari and Barbash, 2011). The tumorigenic genetic interactions involve an oncogene termed *Xiphophorus* melanoma receptor kinase (*xmrk*), which is a mutant duplicate of the fish epidermal growth factor receptor (*egfr*) gene, and a co-evolved suppressor gene, *R(Diff)*, that prevents the oncogene from inducing tumors in the JpA parental line (Wittbrodt et al., 1989). Segregation of these two unlinked loci into backcross hybrids leads to spontaneous tumorigenesis (Schartl and Walter, 2016; Lu et al., 2017) in 50% of backcross hybrids inheriting *xmrk-*driven macromelanophore pigmentation pattern. The *xmrk* oncogene has been shown to drive the dedifferentiation and proliferation of the neural crest derived melanocyte lineage (Wellbrock et al., 2002). The *xmrk* oncogene, as well as the *Sd* (JpA) and *Sp* (JpB) pigment patterns, are X-linked and map to a small region of the X chromosome making recombination between *xmrk* and either *Sp* or *Sd* a very rare event. Therefore, *Sd* and *Sp* are hypothesized to control neural crest lineage migration to specific body compartments, wherein *Sd* migration is to the dorsal fin body compartment, while *Sp* directs neural crest cell migration to the body flanks. Under this hypothesis, the pigmentation pattern of *X. maculatus* is primarily a matter of inheriting either X*^*Sd–xmrk*^* or X*^*Sp–xmrk*^.*

*Xiphophorus* is also a great system to better understand how the risk factors impact the initiation and progression of the disease, allowing studies of interaction between genome and environment. Unlike spontaneous tumorigenesis observed in interspecies hybrids between JpA and *X. hellerii*, a similar interspecies backcross involving JpB as the non-recurrent parent leads to hybrids displaying enhanced macromelanophore pigmentation on the flanks of the animals. Only if interspecies hybrids from the later cross [i.e., *X. hellerii* × (JpB × *X. hellerii*)] are exposed to UVB (or MNU) at juvenile stage do they exhibit induced tumorigenesis at 4 to 6 months of age (Nairn et al., 1996b), similar to human (Craig et al., 2018). In this model, melanomagenesis is a result of interaction between the hybrid genetic background and the environment. Therefore, the etiology of tumorigenesis within interspecies hybrids controlled by the JpA or JpB parental genomes cannot be simply explained by inheritance of X*^*Sd–xmrk*^* or X*^*Sp–xmrk*^*.

We hypothesize that fixed genetic variants between the highly inbred *X. maculatus* JpA and JpB parental lines account for differences in transcriptional phenotypes (i.e., the basal level gene expression landscape), and these differences are related to, and may determine, the tumorigenic trajectory upon interspecies hybridization. Identifying inter-strain genetic variants leading to differences in tumor etiology will forward our molecular genetic understanding of spontaneous and induced melanoma. The comparative genomic and transcriptomic analyses showed that baseline genetic differences involved with inter-cellular and cell-microenvironment interactions. Most importantly, genes encoding core enzymes within base excision repair pathways are enriched with inter-strain genetic variants and differentially expressed genes.

## Materials and Methods

### Animal

Animal use was approved by the Texas State University Institutional Animal Care and Use Review Board (IACUC protocol #2015107711). Fish utilized in this study were maintained in accordance with the applicable OLAW guidelines governing animal experimentation in the United States, and international legislation regulations governing animal experimentation.

*Xiphophorus* utilized in this study were bred and maintained in the *Xiphophorus* Genetic Stock Center^1^. *Xiphophorus maculatus* (*X. maculatus*) JpA and JpB strains used for genome re-sequencing were at their 116th and 109th generation of inbreeding, respectively.

### DNA and RNA Isolation

For DNA isolation, 4 male JpA and 4 male JpB fish were sacrificed by over-anesthetization with MS222 (0.06%). Fish tissues were digested with Proteinase K at 37°C for 1 hr. The lysate was then used for DNA isolation and purification using DNeasy Blood & Tissue Kit (Invitrogen).

For RNA isolation, fish were anesthetized in an ice bath and upon loss of gill movement were sacrificed by cranial resection. Skin and liver tissues were dissected directly into TRI reagent (Sigma Inc., St Louis, MO, United States) and flash frozen in an ethanol dry ice bath. For JpA, skin samples (*n* = 25), and liver samples (*n* = 6) were collected; For JpB, skin (*n* = 30) samples, and liver samples (*n* = 4) were collected for RNA isolation and sequencing library preparation. RNA isolation was performed following the Qiagen RNeasy RNA isolation protocol (Qiagen, Valencia, CA, United States). Tissue samples harvested from fish were first homogenized using a hand-held homogenizer in a 1.5 mL microcentrifuges tube while the sample remained frozen in TRI Reagent (Sigma Inc., St Louis, MO, United States). After homogenization, 300 μL of fresh 4°C TRI Reagent was added to the samples followed by room temperature incubation for 5 min. Chloroform extraction was performed by adding 120 μL chloroform and shaken for 15 s. Samples were centrifuged (16,100 rcf for 5 min at 4°C) for phase partitioning. The aqueous layer was transferred to a new 1.5 mL microcentrifuges tube and a second chloroform extraction performed (300 μL TRI Reagent, 60 μL chloroform). After extraction, nucleic acids in the aqueous phase were precipitated with 500 μL 70% EtOH in diethylpyrocarbonate (DEPC) treated water. The sample was then transferred to a Qiagen RNeasy mini spin column and on-column DNase treatment was performed for 15 min at 25°C. RNA samples were then washed and eluted in 100 μL RNase free water. RNA concentration was measured with a Qubit 2.0 fluorometer (Life Technologies, Grand Island, NY, United States). To further assess the RNA quality, an RNA integrity (RIN) score was determined using an Agilent 2100 Bioanalyzer (Agilent Technologies, Santa Clara, CA, United States). All samples processed for RNA sequencing had a RIN score above 8.

### Genome and Transcriptome Sequencing

DNA samples were forwarded for genome shotgun sequencing library preparation using Illumina Nextera sequencing Library Prep Kit, followed by sequencing on HiSeq 2000 (Illumina, Inc., San Diego, CA, United States) using 150 bp paired-end (PE) sequencing strategy with an average of 33 X genome coverage. Isolated RNA samples were forwarded for Illumina High-throughput Sequencing using the Illumina TruSeq mRNA Library Prep Kit on the HiSeq 2000 platform (Illumina, Inc., San Diego, CA, United States). Each RNA sample was used to construct a single library for sequencing (PE sequencing; See Supplementary Table 7 for details). For both genomic and transcriptomic sequencing, raw reads were trimmed and filtered using a custom Perl script and adapter sequences were removed from the sequencing reads (Garcia et al., 2012). The reads were truncated based on similarity to library adaptor sequences using custom Perl scripts (Garcia et al., 2012). Then, low-scoring sections of each read were removed, preserving the longest remaining sequencing read fragment. Sequencing statistics are included in Supplementary Table 7.

### Identification and Annotation of Genetic Variants

Filtered genome sequencing reads were mapped to the reference genome (X.mac V5.0; NCBI accession number: GCA_002775205.2) using Bowtie2 “head-to-head” mode (Langmead and Salzberg, 2012). Alignment files were further filtered by keeping reads alignments with a MAPQ score ≥30, and sorted using samtools (Li et al., 2009; Li, 2011). Subsequently, pileup files were generated for each *X. maculatus* sample and variant calling was processed by BCFtools and VarScan for SNP and Insertion/Deletion detection, with minimum variant locus coverage of 2 and a *p*-value for variant detection of 0.05 for VarScan, and variant genotyping call Phred score of 0 and alternative genotyping Phred score ≥20 for BCFtools (Koboldt et al., 2009). Only variants that were identified by both pipelines were forwarded for further analyses.

To localize fixed variants between the two inbred *X. maculatus* lines, homozygous loci of JpA were compared to those of JpB. Such loci were identified if ≥75% of JpA is homozygous for one allele, and ≥75% of the JpB is homozygous for another allele. These fixed genetic variants were functionally annotated using snpEff. A genome database was created using the *X. maculatus* genome sequence and annotation files. Each variant was queried to the genome database to determine if it was located in a genic or intergenic region, and to determine what effect each variant may have on the peptide sequence structure. We focused on genetic variants that led to conservative in-frame insertion/deletion, disruptive in-frame insertion/deletion, frame-shift, missense, start codon change, stop codon loss/gain, splicing pattern/exon usage alteration for further biological function and pathway analyses.

For higher stringency in detecting genetic variants, RNA-Seq reads from skin and liver were also mapped to the *X. maculatus* genome using Tophat2 (Kim et al., 2013), followed by sequence variant detection using the same method described above. Only variants that were supported by both DNA and RNA sequencing were kept as inter-strain variants for further analyses. Therefore, all genetic variants were supported by both genome re-sequencing and transcriptome sequencing data.

### Tumor Incidences Comparison

Animal records for scheme A and B interspecies backcross hybrids were collected from the *Xiphophorus* Genetic Stock Center. For both crossing schemes, backcross interspecies hybrids exhibiting *xmrk* linked macromelanophore pigmentation patterns were recorded. From November 2008 to December 2019, there were a total of 290 scheme A hybrids at the XGSC that inherited *xmrk-Sd* (123 exhibited spontaneous tumorigenesis). From January 2010 to June 2019, there were a total of 203 scheme B hybrids that inherited *xmrk-Sp* (11 of them developed spontaneous tumors). A contingency table was tested using animal numbers for each cross scheme that developed tumors, and only exhibited benign pigment cell hyperplasia. A Chi-square test was used to test if the tumor incidences of both cross schemes are independent.

### Differential Gene Expression (DEG) Patterns

Processed transcriptomic sequencing reads were mapped to the *X. maculatus* genome version 5.0 using Tophat2 (Kim et al., 2013), and gene expression of gene models annotated by NCBI was quantified using FeatureCount (Liao et al., 2014). Differentially expressed genes (DEG) between the 25 JpA and 30 JpB were identified using R/Bioconductor package edgeR (Robinson et al., 2010). The DEG test was performed between *X. maculatus* JpA and JpB basal level gene expression. Genes with Log_2_Fold Change (Log_2_FC) ≥1, or ≤−1, False Discovery Rate (FDR) <0.05, with receiver operating characteristic (ROC) curve area under curve (auc) ≥0.8 were determined to be DEGs in skin between the two fish lines. The test was performed using JpA as the control to calculate relative gene expression between the two strains; therefore, if Log_2_FC ≥1, JpB showed higher-expression of a particular gene, or, if Log_2_FC ≤−1, JpA showed higher-expression of a gene.

### DEG Functional Analyses

Sequence homologies between *Xiphophorus* and human were identified using blastn (Shen et al., 2013). A Reciprocal Best Hit (RBH) method was used to identify human orthologs of *Xiphophorus* genes. DEGs between *X. maculatus* JpA and JpB skin, and strain-specific alleles were converted to human homologs and were further submitted to Ingenuity Pathway Analysis (IPA, Qiagen, Redwood City, CA) for functional analyses. Bioinformatics analysis was performed using IPA for clustering and assessing the biological function of DEGs. Herein, the term “pathways” is short for canonical pathways as assigned by IPA based on input genes. Pathway analysis was performed by testing the over-representation of genes belonging to a certain pathway in the input gene list using Fisher exact test. Pathways with an enrichment -log_10_(*p*-value) score >3 (*p*-value < 0.001) were kept for further analysis.

### Principle Component Analyses

Principle Component Analyses (PCA) was performed using R function prcomp. Library size normalized gene expression values were further scaled and subsequently used for PCA. Plot of the first two dimensions were made using custom R scripts.

### Data Visualization

Heatmaps, dot plots and chromosomal plots were made using custom R scripts. All scripts are available upon request.

## Results

### Interspecies Hybrids Exhibit Strain-Specific Tumor Etiology

Spontaneous tumor incidences of two interspecies crosses established between *X. hellerii* and *X. maculatus* JpA or JpB [i.e., *X. hellerii* x (JpA × *X. hellerii*), or scheme A; *X. hellerii*× (JpB × *X. hellerii*), or scheme B] strains were first compared. From November 2008 to December 2019, there were a total of 290 scheme A hybrids at the XGSC that inherited *xmrk-Sd*, and 123 of them exhibited spontaneous tumorigenesis (tumor incidence = 42.4%). From January 2010 to June 2019, there were a total of 203 scheme B hybrids that inherited *xmrk-Sp.* 11 of them developed spontaneous tumors (tumor incidence = 5.4%; Table 1). Scheme A tumor incidence follows Mendelian segregation of an unlinked *xmrk* oncogene modifier, *R(Diff)* (*X*^2^ = 3.1, df = 1, *p*-value = 0.08). In contrast, scheme B tumor incidence does not follow Mendelian distribution (*X*^2^ = 98.4, *df* = 1, *p-*value < 2.2 × 10^–16^). In addition, the genetic mechanism underlying both cross schemes is statistically independent (*X*^2^ = 98.5, *df* = 1, *p-*value ≤ 2.2 × 10^–16^; Table 1).

**TABLE 1:** Scheme A and B backcross hybrid spontaneous tumor incidence.

Scheme A (*X. hellerii* × (JpA × *X. hellerii*)		

*xmrk-Sd* non-tumor	*xmrk-Sd* tumor	incidence
167	123	42.4%

**Scheme B (*X. hellerii* × (JpB × *X. hellerii*)**		

***xmrk-Sp* non-tumor**	***xmrk-Sp* tumor**	**incidence**

192	11	5.4%

### Genetic Variants Between *X. maculatus* JpA and JpB Strains

**FIGURE 2:**
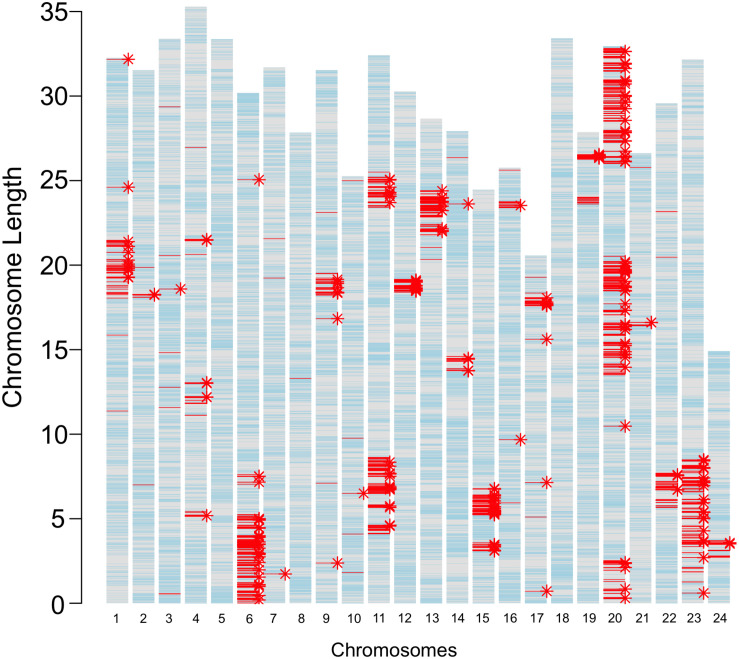
Genetic variants between JpA and JpB strains. The bar plot represents locations of inter-strain genetic variants. The length of each bar corresponds to chromosomal length, with each tick hallmarking 5 Mbp genomic regions. Light blue lines mark annotated gene models; red lines (3,292) highlight fixed genetic variants between the two strains; and asterisks indicate genes that have different coding sequences in the two strains. A total of 3,292 polymorphisms between the two strains were identified. A total 394 of these variants were predicted to change codons of 244 genes.

The JpA and JpB strains were in their 116th and 109th generation of inbreeding, respectively (Figure 1). Using both genome re-sequencing data on 4 JpA and 4 JpB and RNA-Seq data of 25 JpA and 30 JpB samples, we observed 3,292 homozygous polymorphic sites between the JpA and JpB genomes (Supplementary Table 1). The majority of these genetic variants are located within non-coding regions, or located in a coding region, but are not expected to lead to codon changes (i.e., synonymous variants; Supplementary Table 1). However, we identified 393 variants that are expected to produce conservative in-frame insertion/deletion (amino acid change predicated to have minimal effects on protein product), disruptive in-frame insertion/deletion (amino acid change that is predicated to have large effect on protein product), frame-shift, missense, start codon change, stop codon loss/gain, splicing pattern/exon usage alterations, and are therefore predicted to change encoded polypeptide sequences and potentially molecular functions. These 393 variants are located within 244 genes (Figure 2 and Supplementary Table 2). In addition, genetic variants are not randomly distributed throughout the genome, but instead show defined clustering into chromosome “blocks” (Figure 2).

### Gene Expression Landscape Differences Between *X. maculatus* JpA and JpB

**FIGURE 3:**
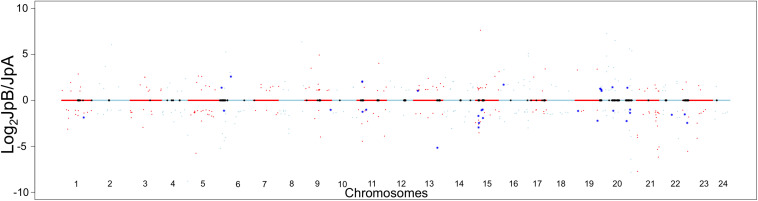
Transcriptional landscape differences modulated by inter-strain genetic polymorphisms Chromosome dot plot is used to present physical locations of genetic variants and inter-strain differentially expressed genes. Relative gene expression in Log_2_ JpB expression/JpA expression were plotted against the chromosomal location of DEGs between JpB and JpA skin. Red and light blue colors hallmark genes located on odd and even number chromosomes, respectively. Black asterisks on the centerlines highlight genes with fixed genetic variants located within the coding regions. Dots highlighted by dark blue asterisks are DEGs that are close (i.e., less than 3 kbp away) to identified genetic variants.

The inter-strain genetic variants are hypothesized to be heterozygous among the ancestral population that gave rise to both strains. These variants, shaped by evolutionary mechanisms (e.g., natural selection, genetic drift and demographic processes), may lead to functional divergence of strain-specific alleles (Wray et al., 2003; Wittkopp, 2006; Wray, 2007; Emerson and Li, 2010). Therefore, we sought to assess transcriptional differences between these two *Xiphophorus* strains in order to infer functional divergence caused potentially by the genetic variants. When comparing transcriptional profiles between the two strains, 412 genes (237 highly expressed in JpA, 175 highly expressed in JpB) were identified to be differentially expressed in the skin (Log_2_FC) ≥1, or ≤−1, FDR < 0.05, ROC curve AUC ≥0.8; Figure 3 and Supplementary Figures 1–3 and Supplementary Table 3).

Using a 3 Kbp length up- or down-stream of the DEGs to estimate *cis-*effects of polymorphisms of gene expression, we found 163 polymorphisms (5%) are adjacent to 32 DEGs (7.8%) in the skin (Figure 3 and Supplementary Table 4).

### Functional Differences Between JpA and JpB Transcriptomes

Next, we investigated functional differences between JpA and JpB strains using observed genomic and transcriptomic divergence between the two fish strains. Both genetic variants (i.e., genes exhibiting coding sequence differences between JpA and JpB), and DEGs between the two strains, were used together to test over-representation of specific signaling pathways (Supplementary Figure 4; pathway enrichment analyses *p*-value < 0.001). This strategy was employed to identify pathways that may include genes exhibiting similar expression levels, but differentiated function due to codon changes, and genes that showed diverged expression patterns. Signaling pathway enrichment analyses were performed by comparing each dataset to databases consisting of common signaling pathways and genes.

**FIGURE 4:**
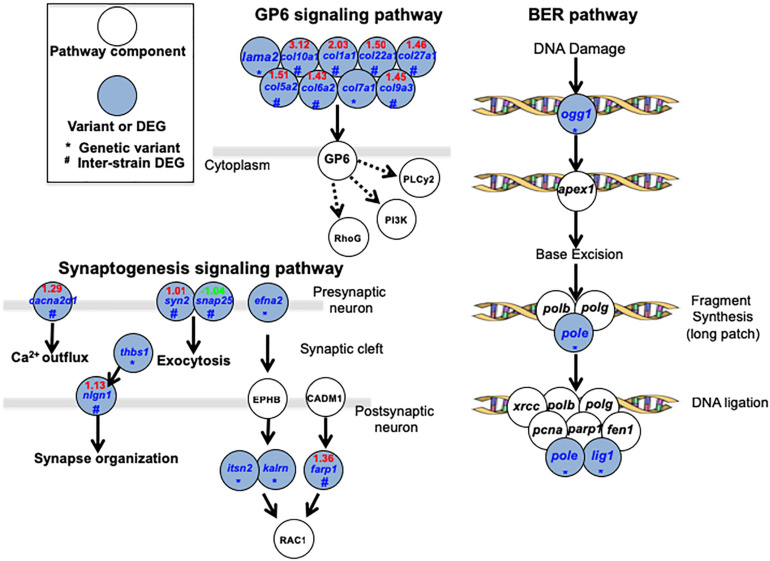
Signaling pathways over-represented by genetic variants and differentially expressed genes Over-represented pathways and related genes are plotted. Blue highlighted genes indicate the presence of a pathway component from either the genetic variants data set or DEG dataset.

A total of 3 major pathways (GP6 signaling pathway, Synaptogenesis signaling pathway and Base Excision Repair pathway; Figure 4) were identified (for specific pathways, see Supplementary Table 5). In GP6 signaling pathways, JpA and JpB genetic differences are represented by laminin and multiple collagen genes. Products of these genes play fundamental roles in establishing the extracellular microenvironment, and differences in expression levels and sequences between JpA and JpB may suggest fundamental microenvironmental differences exist. Synaptogenesis signaling pathways are enriched by genes that are involved in Ca^2+^ outflux (*cacna2d1*), neurotransmitter exocytosis (*syn2* and *snap25*), synapse organization (*thbs1* and *nign1*), and RAC1 mediated effect (e.g., cytoskeletal organization, cell growth, glucose uptake) regulated by *efna2, itsn2, kalrn*, and *farp1* (Ridley, 2006). Another over-represented functional category involves core enzymes of BER genes (i.e., *pole, lig1*, *ogg1*), suggesting JpA and JpB exhibited diverged DNA repair functionality or repair efficiency (Figure 4).

## Discussion

In this study we aimed to dissect the genomic and transcriptomic variants between JpA and JpB in order to interpret functional differences between two *X. maculatus* strains that exhibit diverged tumorigenic trajectories upon inter-species hybridization with other *Xiphophorus* species. Previous studies that utilized backcross hybrids between JpA or JpB, and another *Xiphophorus* parental species to induce tumorigenesis have revealed the locations of tumors to be strongly associated with the macromelanophore pigmentation pattern (i.e., tumors in JpA derived interspecies hybrids located on dorsal fin, tumors in JpB interspecies hybrids located on side of the body) (Setlow et al., 1993; Nairn et al., 1996a; Walter and Kazianis, 2001; Mitchell et al., 2010; Patton et al., 2011; Lu et al., 2017). Therefore, it is hypothesized that genes involved in these pigmentation patterns (i.e., *Sp* or *Sd*) may be involved in the tumorigenic etiology of these two strains. However, differences in scheme A and B spontaneous cancer incidences support the concept that tumorigenesis in JpA and JpB backcross hybrids may occur by two different mechanisms independent of *Sp/Sd* (Table 1).

Comparative genomic data between JpA and JpB strains showed differences between the two strains are not limited to *Sp*/*Sd* loci that are both mapped to chromosome 21 (Gutbrod and Schartl, 1999; Woolcock et al., 2006), but they also exhibit fixed variants in many other loci (Figure 2). Surprisingly, these variants are not distributed randomly on the chromosomes, but rather cluster into large, but discrete genomic regions (Figure 2). Genetic variant density between *Xiphophorus* species, e.g., *X. maculatus* and *X. hellerii*, is 1 per 87 bp (1 SNP per 108 bp, and 1 InDel per 1,067 bp) (Shen et al., 2016). This density is much higher than the inter-strain variant density (i.e., 1 per 16,964 bp) for all chromosomal clusters (Supplementary Table 6). Considering that JpA and JpB strains originated from a single female caught in the wild, it is speculated this founding female and the unknown paternal fish, may have possessed different ancestral alleles present in the wild population. Therefore, the chromosomal distribution patterns of these inter-strain genetic variants may reflect the patterns of meiotic recombination that occurred prior to the genomes of both strains becoming fixed by inbreeding.

The genetic variants between the two strains define genetic background differences that may interact with the recurrent parental genomes upon interspecies hybridization. Although establishment of current genetic background differences may be due to a random ancestral event, characterization of these differences will allow us to perform genetic analyses (e.g., genetic association studies) to identify loci/allele(s) that contribute to spontaneous versus UV-induced melanoma, and to forward our understanding of genetic and environmental contributions to melanomagenesis.

Spontaneous cancer incidence in JpB established backcross hybrids (i.e., scheme B hybrids) is 5.4%, compared to 42.4% within JpA established hybrids (i.e., scheme A hybrids). Although significantly lower, the scheme B hybrid tumorigenesis is still *xmrk*-dependent as it only occurs in individuals that inherited *xmrk-Sp*. In addition, both JpA and JpB potentially inherited the same *R(Diff)* allele as there are either no genetic variants or significant transcriptional differences within the candidate *R(Diff)* locus (Lu et al., 2017). Therefore, a plausible explanation for the low cancer incidence for scheme B hybrids is the *xmrk-R(Diff)* interaction is further modified by additional loci. The incidence of 5.4% may suggest another two unlinked modifier genes [i.e., polygenic; e.g., *xmrk, R(Diff)*, modifier 1 and modifier 2] contribute to spontaneous tumorigenesis in scheme B hybrids. If this is true, then the fraction of hybrids possessing the particular genetic makeup is expected to be 6.25% (i.e., 0.5^4^) within the backcross hybrid cohort [e.g., *xmrk^*JpB/–*^, R(Diff)^*X. hellerii/X. hellerii*^*, modifier 1*^*X. hellerii/X. hellerii*^*, modifier 2*^*X. hellerii/X. hellerii*^*].

By combining both genomic and transcriptomic differences between the two strains, we attempted to assess synergistic mutation effects, in which an altered gene product, and expression differences may play a role. DNA repair related genes encoding core enzymes in the pathway were over-represented within the dataset, and exhibited highest pathway coverage (Figur 4 and Supplementary Table 5). Previous studies have shown that JpB exhibits two to three-fold lower efficiency in “dark repair” clearance of cyclobutane pyrimidine dimers (CPDs) and 6–4 photoproducts in skin following UVB exposure than JpA (Mitchell et al., 2004). UVB exposure of heavily pigmented scheme B hybrids was shown to increase the tumor incidence to 34.8%, or six-fold of basal incidence (Table 1 and Nairn et al., 1996a). Combining these previous observations with the genetic variants between JpA and JpB strains, it shows the two strains are diverged in DNA repair function, suggesting a partial contribution to the etiology difference between UV-induced and spontaneous tumorigenesis.

Comparative transcriptomics also identified novel pathways that may contribute to scheme A and scheme B tumorigenesis mechanisms such as GP6 signaling within which many cellular microenvironment related genes showed genetic and transcriptional differences (Figure 4 and Supplementary Table 5). The major proteins in the extracellular matrix are collagens (Frantz et al., 2010), and emerging evidences suggest collagens have a high impact in tumorigenesis (Kalluri and Zeisberg, 2006; Kalluri, 2016). The genetic differences of collagen related genes between JpA and JpB can potentially explain the extracellular microenvironment contribution to diverged tumorigenesis mechanisms. The synaptogenesis signaling pathway that involves genes mediating inter-cellular interactions (Figure 4 and Supplementary Table 5) was also identified. Although we do not have direct evidence to conclude that synaptogenesis is functionally different between JpA and JpB, it is clear to observe from the genetic data that several aspects related to synapse function are affected (Figure 4). It is worth noting that RAC1 regulations are diverged between the two strains. RAC1 functions are related to glucose transportation, cell growth, cytoskeletal organization, and cell motility (Stallings-Mann et al., 2012; Yang et al., 2012; Xiang et al., 2016). Dysregulation of RAC1 also results in tumor metastasis through epithelial-mesenchymal-transition (Stallings-Mann et al., 2012; Yang et al., 2012). Taken together, the data suggests inter-strain pathway activity differences may collectively predispose cellular transcriptomes into different tumorigenesis trajectory.

In summary, our data suggest that both genomic and transcriptomic divergence have broad functional impacts in two closely related and highly inbred *X. maculatus* strains. With these observations, we conclude that significant differences in transcriptomes between these two closely related genetic lines result in fundamental functional divergence, even between animal lines that may be traced to a single origin.

## Data Availability Statement

The datasets generated for this study can be found in the NCBI GEO (accession number: GSE157537). Link is https://www.ncbi.nlm.nih.gov/geo/query/acc.cgi?acc=GSE157537.

## Ethics Statement

The animal study was reviewed and approved by Texas State University Institutional Animal Care and Use Review Board.

## Author Contributions

YL and RW: acquisition of funds, experiment design, data analyses, and manuscript drafting. TO: data analyses and manuscript drafting. MB: experiment coordination, sample collection, and data analyses. WB: sample collection and data purification. WW: experiment design, sample collection, and data analyses. MS: experiment design, data analyses, and manuscript drafting. All authors contributed to the article and approved the submitted version.

## Conflict of Interest

The authors declare that the research was conducted in the absence of any commercial or financial relationships that could be construed as a potential conflict of interest.

## Footnotes

1 http://www.xiphophorus.txstate.edu

## Supplementary Material

Supplementary Figure 1 Principle Component Analyses of gene expression profiles. The first two dimensions of principle component analyses are plotted. All samples are plotted with colors distinguished different organs, and shapes distinguished different fish strains.

Supplementary Figure 2 Differentially expressed genes between JpA and JpB. Dot plot showing differentially expressed genes. Log_2_FC was plotted against Log_2_CPM difference between JpA and JpB.

Supplementary Figure 3 Expression patterns of differentially expressed genes. Heatmap shows library size-normalized expression (Log_2_CPM) in JpA and JpB skin. Each column represents an individual sample, and each row represents scaled gene expression of a single gene in all samples.

Supplementary Figure 4 Venn diagram showing numbers of differentially expressed genes and variants affected genes. Venn diagram is plotted to show numbers of differentially expressed genes between JpA and JpB skin, genes of different coding sequences between the two strains, and genes that are of both of these categories.

Supplementary Table 1 Summary of genetic variant effects.

Supplementary Table 2 Codons affecting genetic variants.

Supplementary Table 3 Differentially expressed genes between JpA and JpB.

Supplementary Table 4 Genetic variants and their adjacent DEGs.

Supplementary Table 5 Signaling pathways enriched by genetic variants and DEGs between JpA and JpB.

Supplementary Table 6 Genetic variant density of polymorphic chromosomal clusters.

Supplementary Table 7 DNA and RNA sequencing statistics.
