# Supplementary material for: Intra-Strain Genetic Variation of Platyfish (Xiphophorus maculatus) Strains Determines Tumorigenic Trajectory
Source: Front Genet. 2020 Oct 6;11:562594. doi: 10.3389/fgene.2020.562594 (PMC7573281; doi:10.3389/fgene.2020.562594)
Supplement: Supplementary Figure 1 — Principle Component Analyses of gene expression profiles. The first two dimensions of principle component analyses are plotted. All samples are plotted with colors distinguished different organs, and shapes distinguished different fish strains. [file Presentation_1.PPTX]

## Slide 1
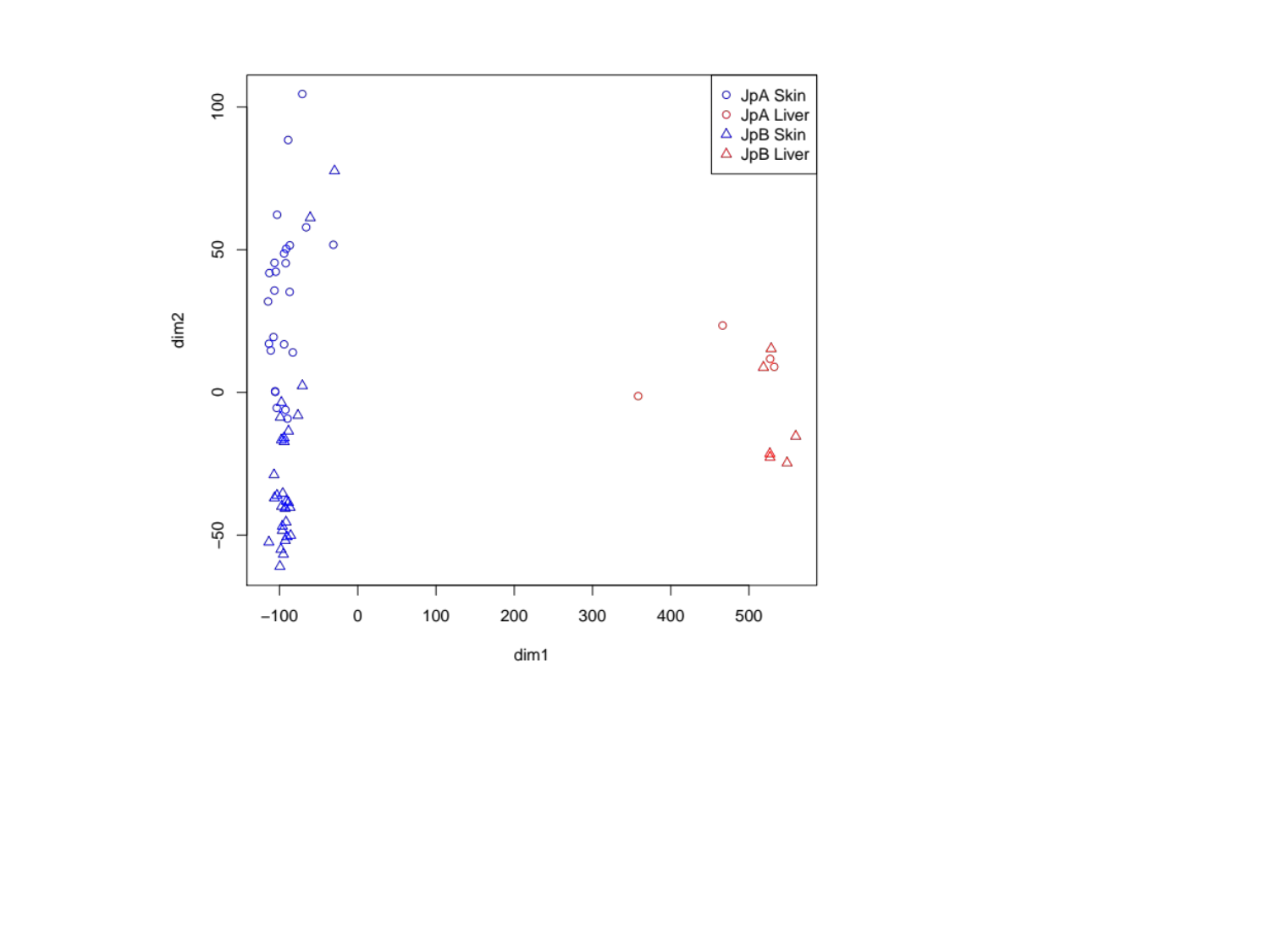

## Slide 2
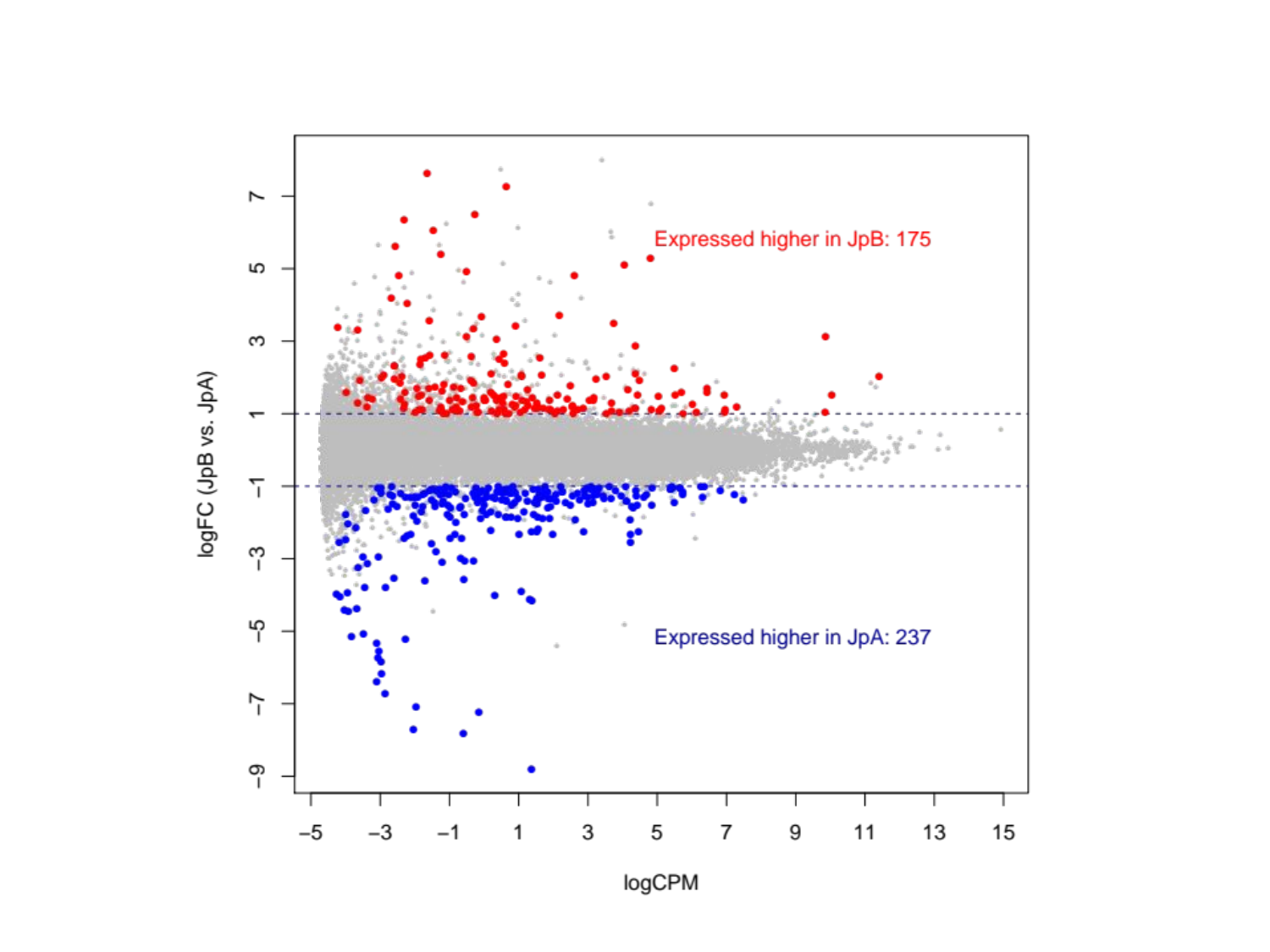

## Slide 3
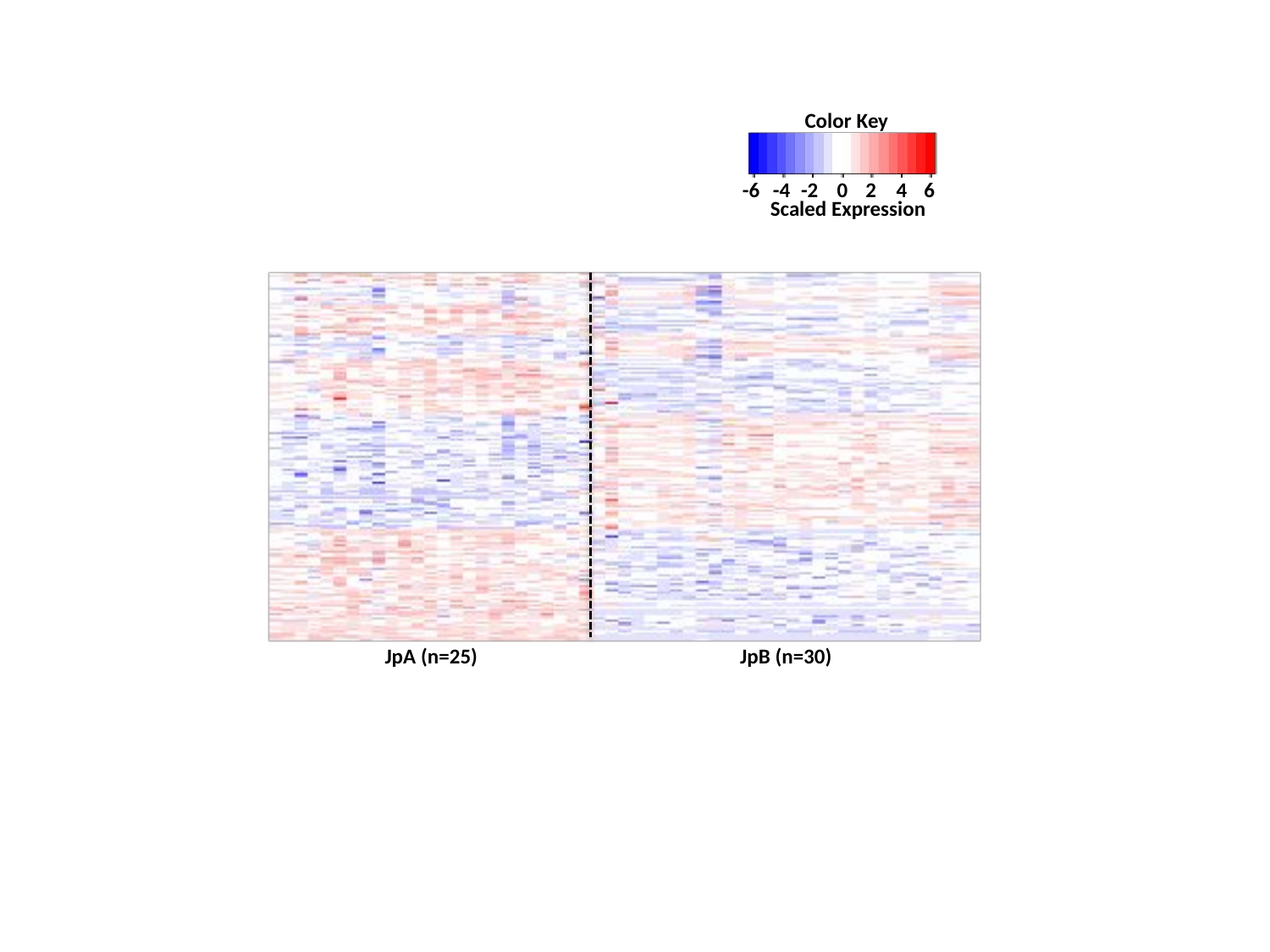

Color Key
6
4
2
0
-4
-2
-6
Scaled Expression
JpA (n=25)
JpB (n=30)

## Slide 4
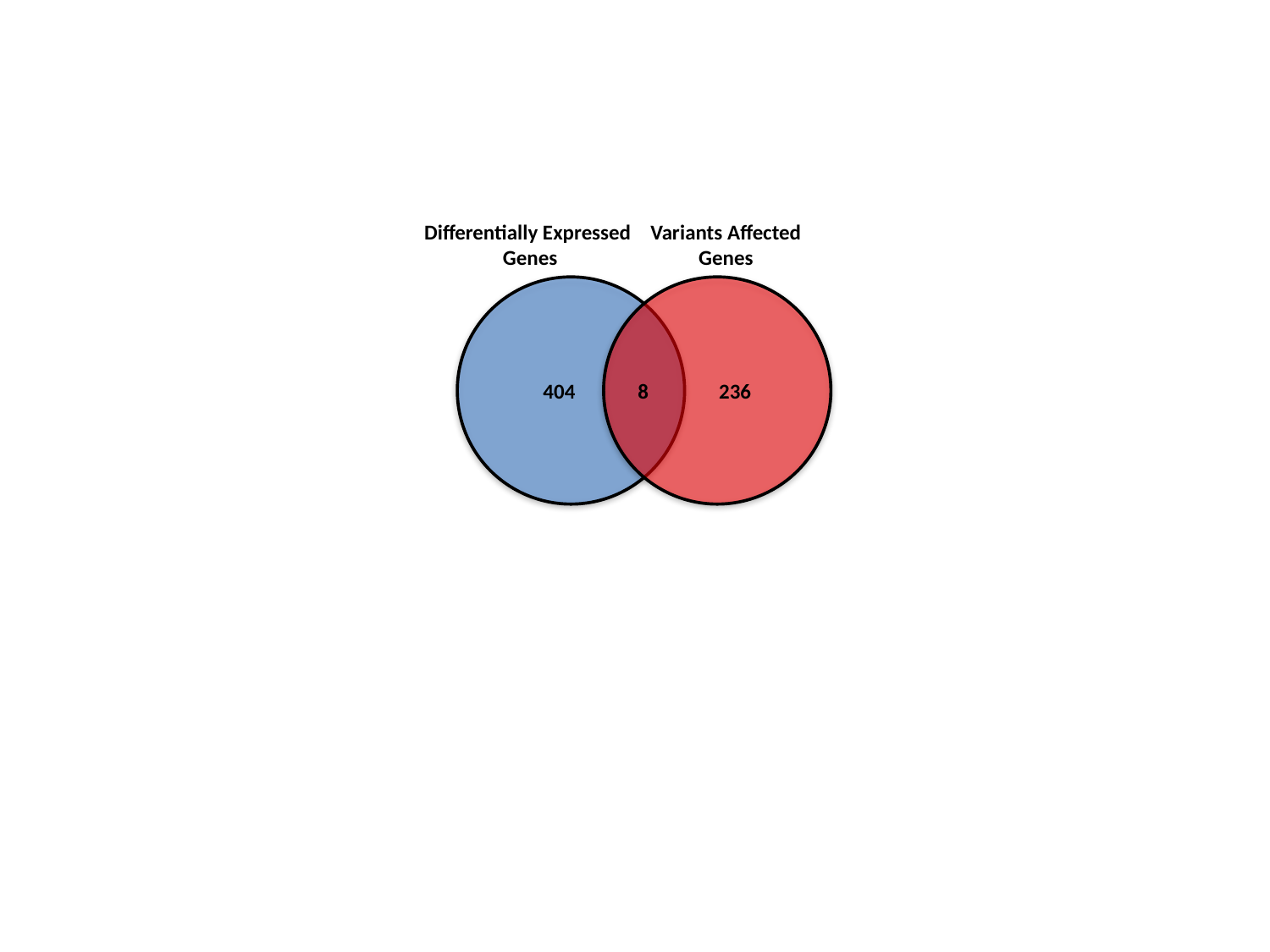

Variants Affected
Genes
Differentially Expressed
Genes
236
404
8
